# Philadelphia Letter

**Published:** 1880-04

**Authors:** 


### Domestic Correspondence.

### ARTICLE XI.

### PHILADELPHIA LETTER.

*Editors of the* CHICAGO MEDICAL JOURNAL AND EXAMINER:

The annual commencement of our two medical schools is a matter of general public interest as well as the event of the year in professional circles. Philadelphia is a small city, whatever may be the number of inhabitants which the census allows her. The intelligent, thinking portion of the community is far more compact and homogeneous than in most American cities of the first class, and any event of an academic nature is a topic of general interest and town-talk. On the morning of commencement, in either of the schools, the neighborhood of the Academy of Music, which is in the center of the city, presents a lively appearance. Flowers in bouquets, baskets, horse-shoes and what-not forms, mysterious packages of various kinds, the anonymous gifts of friends and relatives of the graduates, are handed in at the doors in great quantities. Towards noon the streets in the neighborhood—even gay and crowded Chestnut street, show a more festive aspect, and groups and parties of ladies, all pressing in the same direction, show that an event of general public interest is about to transpire. When one arrives at the door of the building, knots of students are found gathering about while waiting in the large ante-room known as the "foyer," the graduates are assembled, clad in the Oxford academic gown and "mortar-board" hat, and looking, it must be admitted, as well as they ever did in their lives. At one end of the foyer, the trustees, faculty and alumni gather, and there may be seen—I

speak of the University now—a group of prominent and public-spirited men, former merchants for the most part, but men of education whose grey and venerable heads are always to be seen in public assemblies when the common weal is to be forwarded. Probably none of these gentlemen is better known than our late Minister to England, the Hon. John Welsh, whose suave and agreeable presence lends an element of refinement to any gathering. Among the medical men one may see Stillé, whose clear-cut features, high forehead, and white beard, make him a conspicuous object in his velvet gown. I fear this is his last appearance in the procession of commencement day, for I understand that his resignation, presented last year and withdrawn again at the urgent solicitation of the friends of the University, has now been irrevocably determined upon. Agnew, Professor of surgery and rival of Gross in ability as a teacher and in popularity among the students, is also conspicuous from his tall, somewhat stooping figure, fine, bald forehead, surrounded by a fringe of snow-white hair, and with a heavy, white moustache. I believe this exhausts the list of white heads in the University, for we have a young faculty. Pepper, Professor of Clinical Medicine, has a small, light figure, and almost youthful face, of extremely quick and mobile cast, and showing the active brain, never for an instant at rest. Horatio Wood, Jr., is scarcely older-looking, although his full, black beard gives him a more mature look, and, like Pepper, every movement shows the active mind. Looking at him, one can understand the almost morbid craving for work which could induce a man who had written a work on therapeutics, published an investigation on "Fresh Water Algæ of the United States," in the "Smithsonian Contributions to Knowledge," thrown off a "Boylston Prize Essay" and a popular magazine article simultaneously, while at the same time attending to a considerable practice, hospital and private, and delivering a course of lectures as Professor in the University—not to speak of editing a medical journal, to complain bitterly that he was "rusting for want of occupation." Goodell, the obstetrician, whose fine, portly figure and head, with his full brown beard, gains additional picturesqueness from his academic costume, and Norris, the ophthalmologist, are conspicuous figures. Among the other members of the faculty who are in all probability known by reputation to your readers, is Duhring, the dermatologist, and Harrison Allen, professor of physiology, who is the orator of the day—the valedictorian. The procession takes its line of march through the corridors to the stage, and the 111 graduates take their seats in the body of the house, directly in front. Looking from the stage, the sight is a very impressive one; the huge Academy, holding some three thousand persons, is packed tier above tier to the roof, and presents a most brilliant spectacle.

But I will not weary your readers by relating the incidents of a medical commencement, with which they are in all probability extremely familiar. Before the ceremony had begun, I took occasion to scan the list of graduates, and noted the titles of the thesis in each case, marking those which seemed likely to gain distinction. I should say here that various prizes and distinctions, to the number of about sixteen, marked the standing of those who took the honors. I was surprised, when listening to the list of awards, to observe how many of my surmises had proved correct. The subjects of these theses were such as the following: "The Depressor Nerve," "Reparatory Inflammation in Arteries after Ligation," "Physiological Action of Eucalyptol," etc., showing that the effort to study carefully some one subject had resulted in making a worthy contribution to medical knowledge. The unfortunate wight who expects to astonish the world with his original views on "Diphtheria," or "The Origin of Yellow Fever," is apt to be much disappointed at not hearing his name when the honor-list is read at commencement. The valedictory by Prof. Allen was a brief review of the recent progress of medical science, with allusion to the increasing field for public usefulness constantly opening before the profession, as the community at large appreciates more and more the advantage of trained scientific knowledge.

The commencement of the Jefferson Medical College which took place a day or two ago was also a very successful one, although somewhat saddened by the thought that the poet valedictorian of last year, Prof. J. Aitkin Meigs, had since died. A large class, 196 in all, graduated; not quite so many, however, as in previous years. An addition to the faculty has been announced, Dr. Willliam Thompson having been chosen Honorary Professor of Ophthalmology.

The Pennsylvania Hospital situated in the heart of the older part of Philadelphia and worthily renowned for the skill of its surgeons, has always been the center of Philadelphia surgery. It is a pity that more has not come from the advantages enjoyed by the surgeons connected with this hospital, but it must be confessed that except for the admirable clinical lectures which are delivered there twice a week to students from both schools, the profession has not greatly benefitted by the experience of its surgeons. About twelve years ago the publication of a yearly volume of "Hospital Reports" was projected and carried out as far as two volumes, the further publication ceasing for want of funds. The two handsome volumes which remain as relics of this abortive attempt to render the experience of the hospital of general avail, may still be found in the book shops of the city or occasionally bought at auction for a trifle. They contain some very good papers. Recently the surgeons of the hospital have decided to make use of some at least of the valuable material which had accumulated for many years and have published a volume entitled "Surgery of the Pennsylvania Hospital," edited by Drs. Thos. George Morton and William Hunt. This is an octavo of some 350 closely printed pages containing an epitome of the practice of the hospital since 1756; including collections from the surgical notes and an account of the more interesting cases from 1873 to 1878; with some statistical tables. The record of the surgery of the hospital includes 1011 amputations performed in the last fifty years. Of these there were eight of the hip-joint. The rate of mortality for the entire number was 24.43 per cent. The method employed in the treatment of cases, the form of operation preferred, etc., with various tables are given. Following this there are accounts of the various forms of disease and of certain operations performed by the surgeons, giving a mirror of the practice of the institution and containing much that is practical and suggestive. Dr. Morton gives a description of a curious "painful affection of the foot," when the fourth or occasionally the third metatarso-phalangeal articulation is affected by severe pain usually brought on by long walking but occasionally appearing spontaneously, intense in character, "cutting to the heart," accompanied by faintness and cold sweat. There is absolutely no external symptom. Usually the feeling is that of the bones being dislocated but no dislocation can be perceived. Dr. Morton attributes the affection to a bruising of the filaments of the digital branches of the plantar nerve by the contact of the fourth and fifth metatarsal bones.

When this form of neuralgia has been caused by injury, local treatment, rest and anodyne applications may be used; a broad low heeled shoe lacing up the front should be worn. A narrow flannel bandage applied around the foot so as to firmly but moderately support the toe and prevent rolling or friction of the joints is also useful. But in severe cases no treatment except excision of the metatarso-phalangeal articulation, or amputation of the toe with the removal of the joint in question will likely be of any service. The cases subjected to operation all do well and there is no return of the neuralgia. An interesting case where section of the sciatic nerve was performed for elephantiasis arabum of the leg with a certain amount of success is recorded. I am tempted to make a number of excerpts from this volume which is full of interest and practical value, for I think it hardly likely to be seen by many of your readers. But I must content myself by pointing out several features of practical value. The methods of treating fractures employed in the hospital and especially those which are peculiar to it are given at some length and various forms of apparatus are figured. In fact one of the features of the book is the frequent description of procedures peculiar to the locality, "little dodges" and "wrinkles" employed by the surgeons and instruments devised by them. I am not writing a criticism on the the work and so shall not allude to its short-comings. It reminds me of the chat of the consultation room rather than the systematic description of disease and treatment. I venture to call the attention of your readers to the work, being quite sure that it will be found to contain much that is new, and to be rich in practical suggestion.

I believe that in Chicago you are quite ahead of us in all matters relating to public health, and that we have nothing new to tell you upon that point. But it may interest your readers to know that the general impulse which has of late been given to sanitary science has affected the community of this city. Our board of health is almost equally composed of honest hard-working competent physicians and crafty politicians. The result is a constant struggle between the powers of good and evil which is as undecided in its limited sphere as in the world at large. Just now the medical men appear to have the upper hand, and one or two minor reforms have been made which have increased decidedly the comfort and health of our citizens. One of these is the proper cleansing and ventilation of our street-cars. All Philadelphia rides twice a day in the cars which run in all our streets small and large, and that these cars should be decidedly comfortable and healthy is in the highest degree desirable. But there has been no means of ventilating these vehicles until recently a stove has been devised which stands half inside half in front of the car for the comfort of the driver. A constant current of fresh air passes around this stove, heating and ventilating the car at the same time. The time-honored custom of strewing hay to the depth of a foot or more over the bottom of the car has also been stopped by order of the board of health. No one who has not experienced it can guess the disgusting condition of this hay. Trodden under foot by dozens of filthy feet, defiled by constant expectoration of tobacco juice or even of diseased saliva, the mass of reeking foulness must be waded through by all, even refined ladies, to whom it became in time a sort of torture to enter these vehicles. In addition as the augean conglomeration of filth became tramped over, at the end of a trip fresh hay was piled on top, and the rotting mass left to decompose beneath. We thank the board of health for relief from this plague from the bottom of our hearts. A positive measure of public benefit which is also to be carried out is the establishment of public baths in various parts of the city. In a future letter I shall describe these to you at length.

PHILA.

